# In Vitro Percutaneous Absorption of Permeation-Enhancing Estrogen Formulations

**DOI:** 10.3390/ph18040596

**Published:** 2025-04-19

**Authors:** Guiyun Song, Kendice Ip, Bruce Biundo, Maria Carvalho, A. J. Day, August S. Bassani, Hui Song, Benigno C. Valdez, Daniel Banov

**Affiliations:** 1 Professional Compounding Centers of America (PCCA), 9901 South Wilcrest Drive, Houston, TX 77099, USA; 2 Department of Stem Cell Transplantation and Cellular Therapy, The University of Texas MD Anderson Cancer Center, 1515 Holcombe Blvd., Houston, TX 77030, USA

**Keywords:** transdermal, topical, skin, permeation-enhancers, semisolid preparations, estradiol, extemporaneously compounded formulations

## Abstract

**Background/Objectives**: Hormone Replacement Therapy (HRT) is commonly prescribed to women in need to restore the deficiency of hormones. Estrogens, in particular estradiol (E2) and estriol (E3), are associated with side effects when given orally. As such, estrogen is topically applied on the skin for the delivery of the hormone. The objective of this in vitro study is to evaluate the percutaneous absorption of compounded estradiol 0.06% and bi-est E3/E2 0.1%/0.06% in aqueous and anhydrous proprietary permeation-enhancing bases, in comparison with the commercially available estradiol transdermal gel (ESTROGel^®^). **Methods**: The In Vitro Permeation Test (IVPT) was used and validated for the objectives of this study. The strength of estradiol/estriol in five test formulations was determined using Ultra Performance Liquid Chromatography (UPLC). **Results**: ESTROGel exhibited a rapid increase in the rate of skin absorption of estradiol within 0.5 h post-application. This peak was followed by a rapid decline in flux within 4 h, and then a slower decline by 16 h post-application. The initial rapid increase for ESTROGel was much faster than the rate of the four test compounded formulations, which each exhibited a slow and steady increase in the rate of skin absorption of estradiol with a peak flux within 6 h, and a steady absorption within 16 h of application. **Conclusions**: The compounded bases facilitated a steady percutaneous absorption of estradiol, without quick peaking or declining, which is one of the desired characteristics in HRT. Compounding pharmacists and practitioners may consider estradiol compounded formulations as a viable option for hormone delivery to patients.

## 1. Introduction

Hormones are naturally produced chemical substances that work in symphony to control and regulate the body’s functions. Hormonal dysfunction may occur at any age and affect a woman’s overall health and wellbeing. Hormone Replacement Therapy (HRT) may be necessary to restore the hormonal balance ratio, particularly of the sex hormones estrogen, progesterone and testosterone. In the past, regardless of the base-line ratio, women were prescribed the same one-size-fits-all commercial treatments. Medicine has evolved since then and compounded treatments are now customized to meet a woman’s unique hormonal needs [1].

Estrone (E1), estradiol (E2) and estriol (E3) are the main natural estrogens produced by the body. Estrogen deficiency is commonly presented as women grow older, especially E2 following menopause, since this hormone is mainly produced in the ovaries. The decline in E2 may have a profound effect on wellbeing and the associated age-related symptoms including anxiety and depression, urinary tract infections and stress incontinence, osteoporosis, heart disease and cognitive decline [1].

Estrogen Replacement Therapy is commonly prescribed to women in need to re-store the deficiency of the hormones up to optimal, age-appropriate levels. E2 and E3 may be combined according to variable ratios, such as bi-est E3/E2 (80%/20%). Oral estrogen is associated with side effects, and, as such, estrogen is topically applied on the skin using a permeation-enhancing vehicle for the topical delivery of the hormone [1]. In the United States, a transdermal gel for estradiol 0.06% is commercially available as ESTROGel^®^ (ASCEND Therapeutics US, LLC, Herndon, VA, USA). One pump delivers 1.25 g of gel, which contains 0.75 mg of estradiol. ESTROGel is an absorptive hydroalcoholic gel base formulated to provide systemic estradiol replacement therapy by delivering a controlled release of the hormone [2].

The purpose of this study was to evaluate in vitro the percutaneous absorption of permeation-enhancing estrogen formulations, containing estradiol (E2) 0.06% and bi-est, estriol and estradiol (E3/E2) 0.1%/0.06%. Two proprietary, permeation-enhancing compounding bases, an aqueous base and an anhydrous base, were used in this study for the incorporation of the hormones. The extemporaneously compounded estrogen formulations were evaluated and compared with the commercially available estradiol transdermal gel (ESTROGel).

The novelty of this study lies in the testing of innovative compounded bases that may be used to customize Estrogen Replacement Therapy. The current commercially available treatment does not meet the variable, individual needs of women. By comparing the performance of both treatments (personalized and commercial), new insights may be obtained into novel, alternative ways to address estrogen deficiency in women.

## 2. Results

### 2.1. In Vitro Drug Release

**Figure 1:**
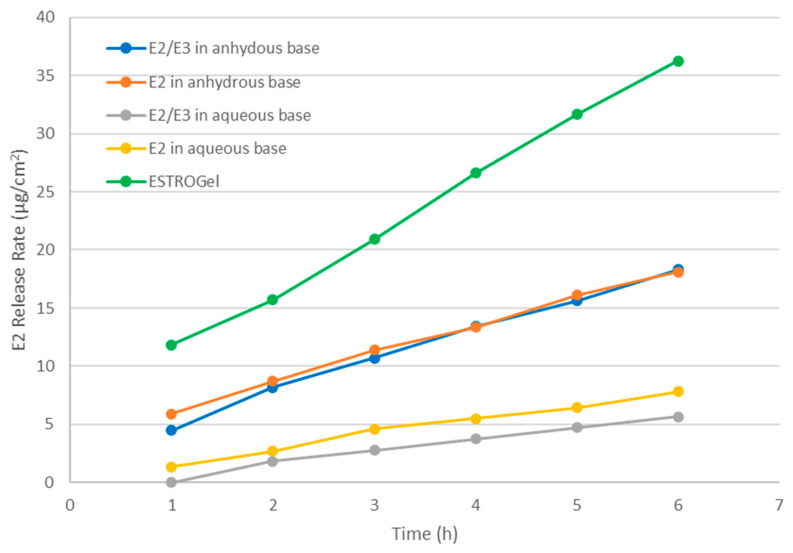
Rate of drug release for estradiol across the five test formulations for 6 h.

The release rate of estradiol across the five test formulations was compared for 6 h, and the highest release was obtained for ESTROGel, as shown in Figure 1. The second highest release rate was achieved by the anhydrous formulations. Both estradiol 0.06% and estradiol 0.06%/estriol 0.1% in the anhydrous base showed a very similar release rate. The lowest release rate was achieved by the aqueous formulations, with both showing a very similar release rate profile (Figure 1).

### 2.2. In Vitro Permeation Test (IVPT) Validation

#### 2.2.1. IVPT Apparatus Qualification

Prior to evaluating the in vitro percutaneous absorption of the estrogens, the apparatus and the percutaneous test were validated to reduce data variability and to enhance reproducibility. Based on the results for apparatus qualifications using various parameters and the predefined acceptance criteria by the USP General Chapter <1724> [3], the apparatus was determined to be suitable for the IVPT method as shown in Table 1.

#### 2.2.2. Solubility and Stability of Estradiol in the Receptor Medium

The measured solubility of estradiol in the receptor medium was 217.54 µg/mL, which was more than 10 times higher than the highest measured concentration in the samples obtained during the method development experiments. These results confirmed that the solubility of estradiol in the receptor medium was sufficiently high to ensure sink conditions.

For the bench-top and long-term stability validations, around 1 µg/mL of estradiol in the receptor medium was used (Table 2). Estradiol was stable in all formulated samples within 24 h at room temperature as determined by UPLC (Ultra-Performance Liquid Chromatography). Similarly, estradiol was stable after 14 days at 80 °C. Sample stability was considered acceptable if the mean measured concentrations of the solutions did not deviate by more than ±15% from the nominal concentration. The results suggest that estradiol is stable at room temperature and −80 °C for 24 h and 14 days, respectively (Table 2).

#### 2.2.3. Dilution Integrity

Since the sample concentration exceeded the ELISA Upper Limit of Qualification (ULOQ), it was necessary to dilute the sample with a blank matrix. Dilution integrity was evaluated based on the accuracy and precision of the samples diluted at 5× (times), 50×, 100×, 200× and 300× with the surrogate matrix (ELISA buffer). The accuracies were 82–103% for 5×–200× dilutions (Table 3). Precisions were 4–9% for 5×–200× dilutions. These results satisfied the ±20% acceptance criterion, suggesting that samples with concentrations exceeding the ULOQ may be diluted up to 200× prior to the analysis. The accuracy and precision of the 300× dilution exceeded the acceptance criteria (reject); thus, the samples were not diluted more than 200×.

#### 2.2.4. Sensitivity

**Figure 2:**
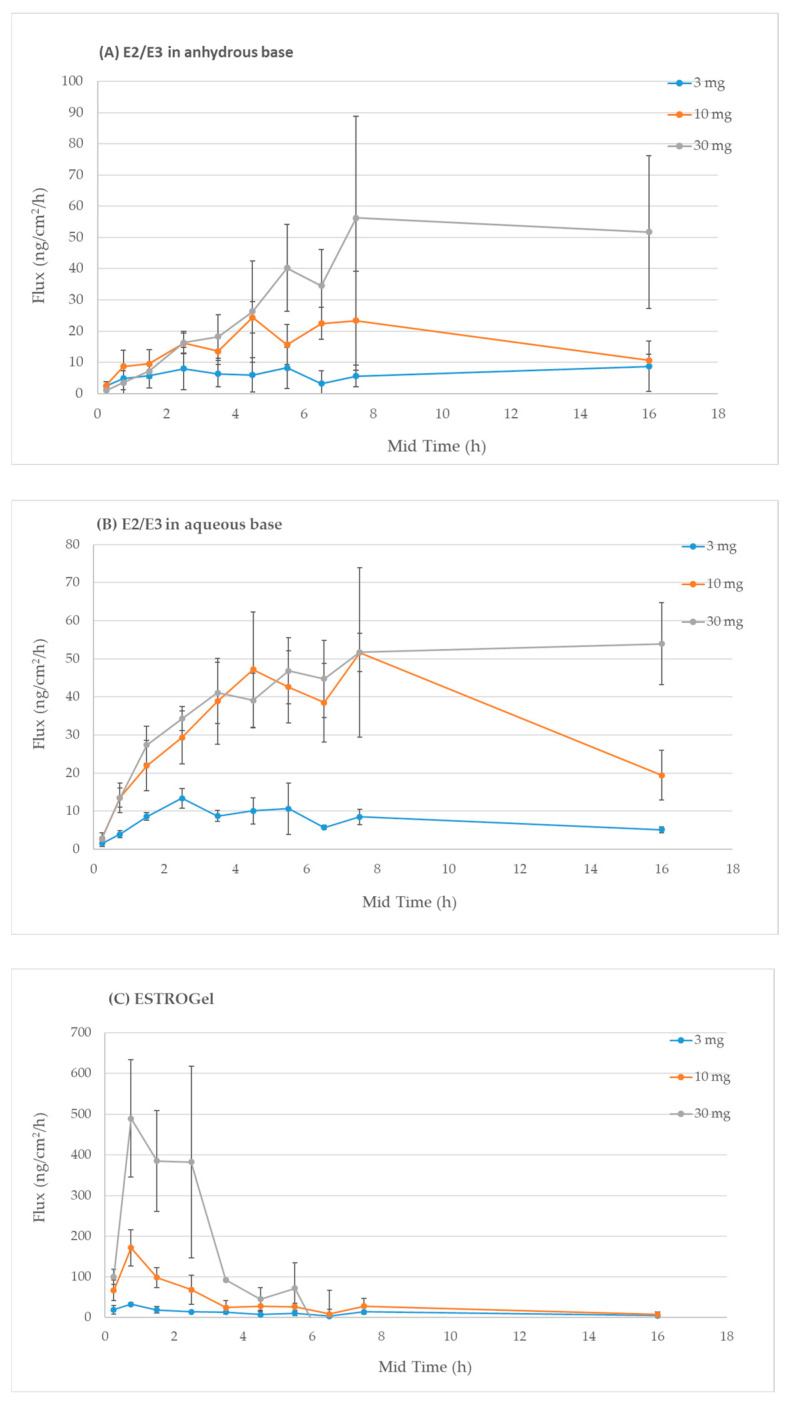
Validation of the IVPT sensitivity using different doses of estradiol inE2/E3 in anhydrous base (**A**), E2/E3 in aqueous base (**B**) and ESTROGel (**C**).

The IVPT sensitivity is the ability of the IVPT method to detect changes in the cutaneous pharmacokinetics of estradiol as a function of differences in estradiol delivery. Figure 2 shows that the mean flux rate of estradiol increased with increasing estradiol formulation doses (3, 10 and 30 mg/cm^2^), suggesting that the IVPT method successfully distinguished the different estradiol doses.

#### 2.2.5. Selectivity

**Figure 3:**
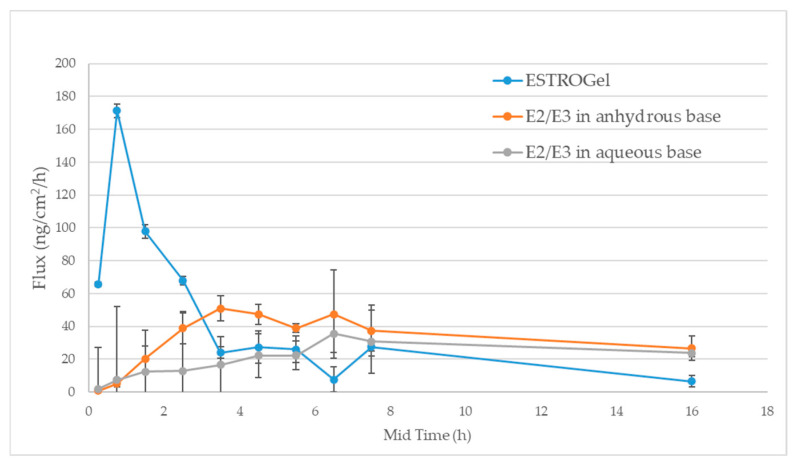
Selectivity analysis for three estrogen test formulations.

The IVPT selectivity determines the ability of the skin permeation method to determine the cutaneous pharmacokinetics of a drug between products (or formulations) that exhibit differences in drug delivery. In this study, the selectivity was evaluated by comparing ESTROGel, E2/E3 in an anhydrous base and E2/E3 in an aqueous base. The cutaneous pharmacokinetics of estradiol in the three formulations exhibited differences in the drug delivery. The results showed that the IVPT methodology is selective for those three test formulations (Figure 3).

#### 2.2.6. Robustness

**Figure 4:**
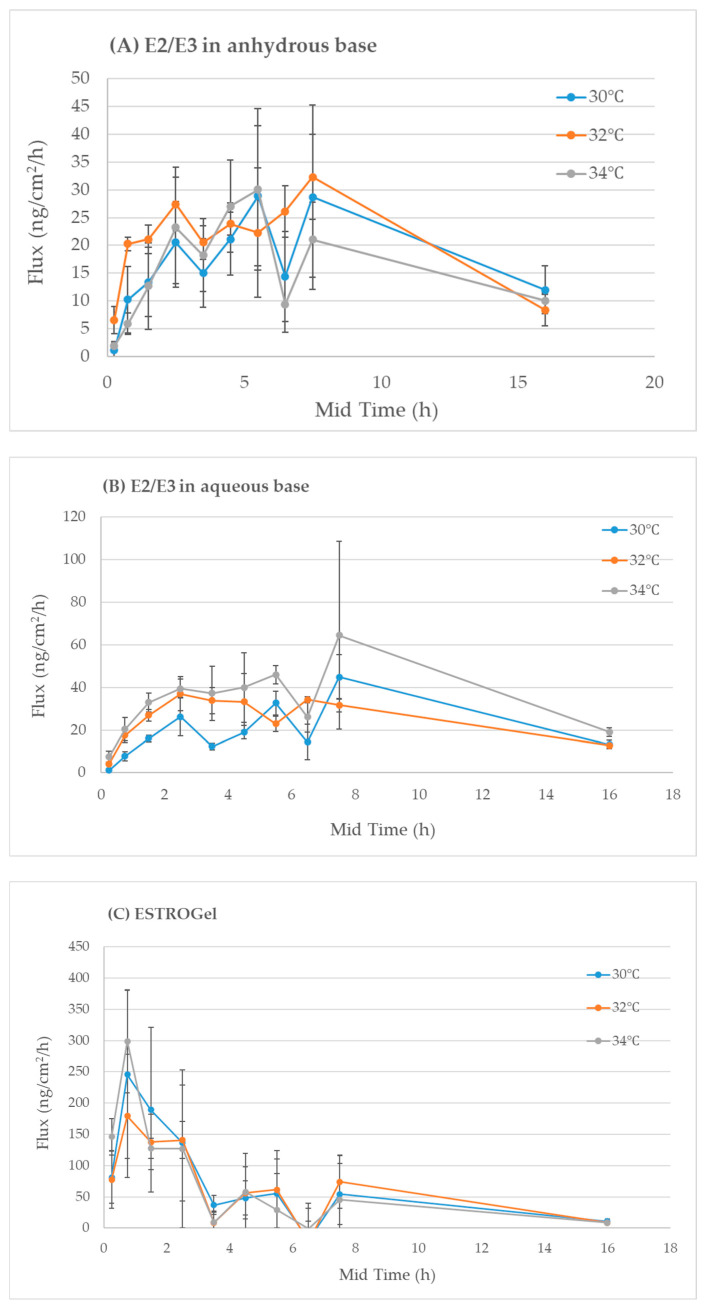
Robustness analysis at various skin surface temperatures using three estrogen test formulations.

The robustness of the IVPT method to minor perturbations in skin surface temperature was evaluated. The mean flux rate of estradiol permeation calculated from the IVPT experiments at 30 °C and 34 °C of skin surface temperature did not deviate from the flux rate of estradiol permeation obtained from experiments using the nominal IVPT method parameters (32 °C). These results confirm that the IVPT method is robust in skin temperature changes between 30 °C and 34 °C (Figure 4).

### 2.3. In Vitro Percutaneous Absorption

The percutaneous absorption of estradiol was compared in vitro using three human donors, three skin samples from each donor and five estrogen test formulations, as follows: ESTROGel (commercial); estradiol 0.06% in the anhydrous base and estradiol 0.06% in the aqueous base (compounded); bi-est E3/E2 0.1%/0.06% in the anhydrous base and bi-est E3/E2 0.1%/0.06% in the aqueous base (compounded). It was observed that the anhydrous and aqueous base formulations provided different profiles of in vitro percutaneous absorption of estradiol compared with ESTROGel.

**Figure 5:**
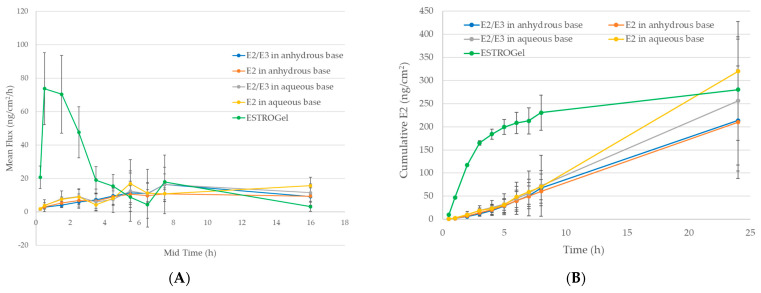
Comparison of skin absorption of estradiol compounded formulations with ESTROGel formulation. The mean flux rate (**A**) and total percutaneous absorption (**B**) of estradiol were determined as described in Methods.

ESTROGel exhibited a rapid increase in the rate of skin absorption of estradiol with a peak flux beyond 70 ng/cm^2^/h within 0.5 h post-application of the test commercial formula. The peak was followed by a rapid decline in flux within 4 h post-application, and then a slower decline in flux until 16 h post-application (Figure 5A). The initial rapid increase for ESTROGel was much faster than the rate of the four test compounded formulations, which each exhibited a slow and steady increase in the rate of skin absorption of estradiol with a peak flux around 10 ng/cm^2^/h within 6 h post-application, and a steady absorption within 16 h of application (Figure 5A).

For the 16 h of study, estradiol in ESTROGel had the lowest flux rate average of 3 ng/cm^2^/h compared with 9–16 ng/cm^2^/h flux rate in the other four formulations. The simple main effects of the applied formulations (aqueous vs. ESTROGel; anhydrous vs. ESTROGel), time and their interactions, were statistically significant (*p* < 0.05) using a two-way ANOVA as shown in Table 4. These results were reflected in the total absorption (cumulative) of estradiol. Initially, ESTROGel provided the highest amount of absorbed estradiol within 8 h of application, but estradiol in the aqueous and anhydrous bases showed a steady increase in its accumulation within 24 h (Figure 5B). After 24 h of application, the total amount of permeated estradiol in the anhydrous base formulation was 109.5 ng/cm^2^ (with estriol) and 121.4 ng/cm^2^ (without estriol); for the aqueous base, the values were 138.5 ng/cm^2^ and 107.1 ng/cm^2^ with and without estriol, respectively (Figure 5B). The average accumulated estradiol using ESTROGel was 116.7 ng/cm^2^. Using a two-way ANOVA analysis, the interactions between the effects of applied formulations (aqueous vs. ESTROGel; anhydrous vs. ESTROGel) and time were not statistically significant (*p* > 0.05) except for E2 in the aqueous base vs. ESTROGel (*p* < 0.05), as shown in Table 4. The simple main effect analysis showed that both applied formulations (aqueous vs. ESTROGel; anhydrous vs. ESTROGel) and time had statistically significant differences (*p* < 0.05) on estradiol permeation (Table 4).

## 3. Discussion

ESTROGel is one of the most popular commercial formulations for the topical application of estradiol. The present study shows that when estradiol is incorporated in either anhydrous or aqueous proprietary compounding bases, it is more steadily delivered through the skin in comparison with the commercial formulation. These results are consistent with previously published studies. Multiple hormones have been shown to be percutaneously absorbed when incorporated in the same aqueous proprietary compounding base [4,5,6,7]. Similarly, in a study by Song et al. [8], the anhydrous base safely and efficaciously delivered progesterone, testosterone, estriol and estradiol through the skin.

It was demonstrated that ESTROGel presents the highest estradiol release rate, resulting in the greatest peak of skin absorption, as opposed to the compounded formulations. In HRT, it may be argued that a steady delivery of hormones is often preferred over rapid fluctuations, as it more closely mimics the body’s natural processes. The differences in drug release and the rate of percutaneous absorption of estradiol 0.06% among the test formulations may be partly attributed to the differences in their excipients. ESTROGel contains ethanol, carbomer 980, trolamine and water. Carbomer 980 is a thickening agent which consists of cross-linked polyacrylic acid that forms a viscous sparkling clear gel with ethanol and water. Trolamine or ethanolamine is used as a pH adjuster and surfactant, and it improves the solubility of carbomer 980 and estradiol. The aqueous proprietary compounding base consists of a topical cream that simulates the natural moisturizing barrier of the skin through its emulsion system [4,5,6,7]. On the other hand, the anhydrous base includes the ingredients phosphatidylcholine and jojoba esters and is thus likely to provide better solubility for the lipophilic estradiol and enhanced permeation properties [9,10]. Since it is an anhydrous base, it provides an unfavorable environment for microbial growth, contributing to extended default beyond-use dates (BUDs) without compromising drug permeation.

ESTROGel is associated with common side effects such as skin irritation [11]. The safety of the anhydrous proprietary compounding base was recently evaluated using the in vivo skin irritation and sensitization Human Repeat Insult Patch Test with no side effects reported [8]. Although a clinical trial for estradiol delivery using anhydrous and aqueous bases is warranted, the present study provides compounding pharmacists and physicians with an alternative for topical estradiol delivery in HRT.

Limitations of this study include its small number of donor skin samples, which may not be statistically powerful enough to obtain more significant results. Despite the widespread use of the Vertical Franz Diffusion System, this device has some limitations such as static and non-flowing properties, a relatively large solution volume and skin area and the possible production of gas bubbles during sampling. The results presented in this study are a prediction or an extrapolation of the actual skin absorption in humans. Nevertheless, the methodology used provides a conceptual base for future clinical trials. An animal study may not necessarily be relevant due to interspecies differences in skin physiology. The anatomical site, skin hydration and age of the person are important factors that may affect the skin absorption of estradiol and should be considered in the future [12].

## 4. Materials and Methods

### 4.1. Materials and Reagents

Vertical Franz diffusion cells were purchased from PermeGear, Inc. (Hellertown, PA, USA). Phosphate-Buffered Saline (PBS) and gentamicin were obtained from Sigma-Aldrich, Inc. (St. Louis, MO, USA). In addition, 2-hydroxypropyl-β-cyclodextrin and Estradiol ELISA kits were purchased from Cayman Chemical (Ann Arbor, MI, USA). ESTROGel 0.06% was manufactured by DPT Laboratories (San Antonio, TX, USA). Human cadaver skin samples were purchased from BioIVT (Baltimore, MA, USA). Estradiol and estriol formulations in anhydrous and aqueous bases were supplied by Professional Compounding Centers of America (Houston, TX, USA).

### 4.2. Test Formulations

The following extemporaneously compounded formulations were used in the study.

(1) ESTROGel containing 0.06% estradiol (E2)
(2) Estradiol 0.06% (*w*/*w*) (E2) in anhydrous base
(3) Estradiol 0.06% (*w*/*w*) (E2) in aqueous base
(4) Estradiol 0.06%/Estriol 0.1% (*w*/*w*) (E2/E3) in anhydrous base
(5) Estradiol 0.06%/Estriol 0.1% (*w*/*w*) (E2/E3) in aqueous base

The formulations 2 and 4 were prepared by adding the hormone(s) to propylene glycol (10%) and then incorporating this into the anhydrous, proprietary permeation-enhancing base (Anhydrous VersaBase HRT, PCCA, Houston, TX, USA). This anhydrous base uses a unique, patent-pending delivery system designed to improve the solubility of lipophilic molecules, such as hormones, and has been proven to increase the permeation of those molecules into and through the skin. Despite this base being an anhydrous system, these lipophilic hormones are able to partition (release) from it and into the skin [13]. The qualitative list of ingredients of the anhydrous base is as follows: cyclopentasiloxane, caprylyl methicone, PEG-16 macadamia glycerides, polysilicone-11, PEG-12 dimethicone/PPG-20 crosspolymer, 1,2-hexanediol, phosphatidylcholine, jojoba esters, isopropyl jojobate, jojoba alcohol and tocopheryl acetate. This compounding base is described as a faint beige, smooth silicone gel with a characteristic odor, and it has water activity below 0.6 (Aw < 0.6), which classifies it as an anhydrous base [13]. The specifications for viscosity and specific gravity are 17,400–32,500 CPS and 0.90–1.10, respectively.

The formulations 3 and 5 were prepared as well by adding the hormone(s) to propylene glycol (10%) and then incorporating this into the aqueous, proprietary permeation-enhancing base (VersaBase Cream, PCCA, Houston, TX, USA). This aqueous base is an ideal compounding base for topical and vaginal HRT. It simulates the natural moisturizing barrier of the skin through its emulsion system and rubs in quickly, leaving a soft and silky feel [14]. The qualitative list of ingredients of the aqueous base is as follows: water, emulsifying wax NF, ethylhexyl stearate, cyclopentasiloxane, sorbitol, aloe barbadensis leaf juice, glycerin, tocopheryl acetate, lactobacillus ferment, citric acid, disodium EDTA, sodium levulinate, sodium anisate and sodium benzoate. This compounding base is described as a white smooth cream with a characteristic odor. The specifications for pH, viscosity and specific gravity are 4.5–6.5, 49,000–82,000 CPS and 0.90–1.10, respectively.

The strength (potency) of estradiol/estriol in the different formulations was tested using UPLC (Waters Acquity UPLC H-class, Waters, Milford, MA, USA), which was equipped with a separation module, an auto sampler, a column heater/cooler and a Photodiode Array (PDA) detector. The UPLC method utilized a Waters Acquity UPLC BEH C18 column with 1.7 µm particle size, 2.1 mm inner diameter, and 100 mm column length heated at 65°C. Mobile phases were 0.1% formic acid in water and 0.1% formic acid in acetonitrile, with a flow rate of 0.6 mL/min. The elution started at 20% of the organic mobile phase, gradually increased to 25% in 2 min, rose to 60% at 4.5 min of run time, followed by 80% at 6 min, returned to 20% at 6.1 min and remained at 20% from 6.1 to 7 min of run time to equilibrate the column. The auto sampler kept the standard and sample vials at 8 °C. Standards and samples were injected at 4 µL in each injection. Detection and quantification were conducted at 280 nm. The mean percent of labeled concentration was found to be 103.03%, 102.23%, 99.47%/97.3%, 100.07% and 97.33%/97.16% in ESTROGel, E2 in the anhydrous base, E2/E3 in the anhydrous base, E2 in the aqueous base and E2/E3 in the aqueous base, respectively.

### 4.3. In Vitro Drug Release

The in vitro drug release test was evaluated using the Franz Diffusion System (surface area 1.77 cm^2^). The diffusion cells were mounted in a diffusion apparatus including PBS plus 0.01% gentamicin and 0.5% 2-hydroxypropyl-β-cyclodextrin as the receptor medium. The study methodology was adapted from the USP General Chapter <1724> [3]. The receptor medium was degassed by filtering through a 0.2 µm membrane and maintaining a vacuum for 5 min; it was then warmed in a water bath at 32 °C. The dialysis membranes (cut off 12–14 kD) were soaked in water overnight, followed by the receptor medium for 30 min at 32 °C. The dialysis membranes were then mounted on the Franz diffusion cells, and 400 mg of each test formulation was dosed on the donor chamber. The receptor medium solution was stirred magnetically at approximately ~600 RPM with the water jacket temperature controlled to maintain at 32 ± 1.00 °C. The receptor medium samples were collected at 1, 2, 3, 4, 5 and 6 h by stopping the stirrer, withdrawing 1 mL of the sample and replacing the same volume with the receptor medium. All receptor medium samples were filtered with a PVDF membrane prior to the quantification of estradiol by the analytical method UPLC with Ultraviolet PDA.

### 4.4. IVPT Validation

#### 4.4.1. IVPT Apparatus Qualification

The IVPT system used to assess estradiol skin permeation consisted of six vertical diffusion cells (VDCs) with a diffusional surface area of 1.767 cm^2^. The diffusion cells were mounted on a six-station diffusion apparatus equipped with individual stirrer motors, and the cells connected to a Polystat Temperature Controller (Cole-Parmer, Vernon Hills, IL, USA). The actual receptor chamber volume was 12 mL. The capacities and the diameters of the orifices of each VDC required for calculation of the amount permeated per unit area, and sampling time, were determined by weight and length measurements, respectively. Purified water was used to assess the capacity of each VDC by weight. The diameters of the orifice were measured with a calibrated Vernier caliper. The skin surface temperature was maintained at 32 ± 1 °C with a temperature controller. The stirring speed was 600 ± 10 rpm according to the manufacturer’s mechanical report.

All physical parameters were measured in triplicate. Mean ($\bar{x}$) and range of variation (V) were calculated to determine the accuracy and the inter-cell variability of the six VDCs in accordance with the predefined acceptance criteria. The acceptance criteria for the apparatus qualification were based on the USP General Chapter <1724> [3].

The workbench levelness, distance above the apparatus to any cooling vents and any exposure to direct sunlight were evaluated. The workbench levelness was measured in two directions with a digital spirit level, ensuring an inclination of less than ± 1°. Care was taken to ensure that the VDCs were not exposed to direct sunlight or cooling vents. The working environment was controlled at 21 ± 2 °C room temperature and 50 ± 20% humidity.

#### 4.4.2. Estradiol Solubility in the Receptor Medium

The solubility of estradiol in the receptor medium containing PBS plus 0.01% gentamicin and 0.5% 2-hydroxypropyl-β-cyclodextrin was evaluated. Weighed estradiol was placed into a beaker and the receptor medium was added to obtain a saturated concentration. The estradiol solution was stirred at room temperature overnight. Aliquots of the supernatant were filtered and analyzed for the concentration of the dissolved estradiol. Ideally, the solubility of the drugs should be 10 times greater than the maximum expected concentration of the drugs in the receptor medium throughout the IVPT run.

#### 4.4.3. Bench-Top Stability and Long-Term Stability of Estradiol in the Receptor Medium

For the bench-top stability validation, around 1 µg/mL of estradiol in the receptor medium was analyzed by UPLC. The vehicles for test formulations and human skin were added to the receptor medium to mimic the real experimental conditions. The mixtures were incubated in a water bath at 32 °C. Samples were collected at 0, 4, 8 and 24 h, filtered with a PVDF membrane and stored at −80 °C for later analysis. The long-term stability of estradiol in the receptor medium was analyzed with the UPLC method after 14 days of storage at −80 °C.

#### 4.4.4. Skin Qualification

Human female abdominal skin samples at a thickness of 430–600 µm were used in this study. To confirm that the skin tissues were intact prior to dosing, an electrical resistance barrier integrity test was performed using a precision LCR meter set at a low voltage alternating current. Any skin samples exhibiting an electrical resistance < 4 kΩ were rejected and replaced. The electrical resistance cut-off value was in accordance with the published data [5] and considered 4.0 kΩ as corresponding to a tritiated water permeability coefficient of 4.5 × 10^−3^ cm/h [15]. The skin donor demographics are shown in Table 5.

#### 4.4.5. Dilution Integrity

The integrity of the dilution was validated by diluting Quality Control (QC) samples with an ELISA buffer to bring them within the quantitation range. One undiluted sample was analyzed and considered as the ULOQ. The accuracy and precision of these diluted and undiluted QCs were demonstrated. Dilutions used during the validation experiments mimicked the expected dilutions in the study.

#### 4.4.6. IVPT Sensitivity and Selectivity

The sensitivity of the IVPT method was evaluated in three dose amounts (3 mg/cm^2^, 10 mg/cm^2^ and 30 mg/cm^2^) of test formulations including ESTROGel, E2/E3 in an anhydrous base and E2/E3 in an aqueous base. The selectivity of the IVPT method was performed using the same test formulations. Four tissues from the same donor were used for each dose and formulation. Receptor medium samples were collected at 0, 0.5, 1, 2, 3, 4, 5, 6, 7, 8 and 24 h. A 1 mL sample was withdrawn from the receptor chamber for analysis, and 1 mL of the fresh receptor medium was replenished into the receptor chamber. The estradiol concentrations were quantified by ELISA (Estradiol ELISA kit, Item No. 501890, Cayman Chemical, Ann Arbor, MI, USA). The differences in the cutaneous pharmacokinetic profiles were expressed in the flux rate (ng/cm^2^/h).

#### 4.4.7. IVPT Robustness

The robustness of the method was investigated using the test formulations at various skin surface temperatures (30 °C, 32 °C and 34 °C). Four tissues from the same donor were used for each temperature and each formulation. The robustness was evaluated after the calculation of the responses to various temperatures. The responses were expressed in the flux rate (ng/cm^2^/h).

### 4.5. Assessment of Differences Between Commercial and Compounded Formulations

The percutaneous absorption of estradiol in the five formulations was determined using three donated female abdominal skin samples following the same methodology as in the validation experiments. The skin surface temperature was maintained at 32 °C.

### 4.6. Statistical Analysis

A two-way ANOVA was used to determine statistical differences among the mean values of the flux rate and the cumulative amount of estradiol transported through the skin at each time point across test formulations and skin donors. Statistical analyses were performed using Analysis ToolPak in 2016 Excel. *p* values less than 0.05 were considered statistically significant. All results were expressed as mean ± SD of treatments in triplicates or quadruplicates.

## 5. Conclusions

The proprietary aqueous and anhydrous permeation-enhancing bases described in this study provide an efficient delivery of estradiol through the skin. The compounded bases facilitate a steady percutaneous absorption of estradiol, without quick peaking or declining, which is one of the desired characteristics in HRT. Based on these results, compounding pharmacists and practitioners may consider estradiol compounded formulations as a viable option for hormone delivery for patients undergoing HRT.

## Figures and Tables

**Table 1:** Predefined acceptance criteria and results for apparatus qualifications.

Parameter	Acceptance Criteria	Results	Range of Variation V	Pass
	Mean	Mean		
Capacity of the cells	12 ± 0.6 mL	11.93 mL	0.4 mL	Pass
Diameter of the orifice	15 ± 0.75 mm	14.7 mm	0.1 mm	Pass
Temperature of the skin surface	32 ± 1 °C	31.7 °C	0.6 °C	Pass
Speed of the magnetic stirrer	600 ± 60 rpm	600 ± 10 rpm	NA	Pass
Dispensed sampling volume	1000 ± 30 µL	1002 µL	6.4 µL	Pass

NA: not applicable; data were obtained from the manufacturer’s instructions.

**Table 2:** Predefined acceptance criteria and results for bench-top and long-term stability for estradiol.

Parameter	Acceptance Criteria	Results				
Bench-top	The accuracy (nominal) at each level should be ±15%	Formulation	Concentration (µg/mL)	0 h %	24 h %	Pass
		ESTROGel	1.64	100.00	101.57	Pass
		E2/E3 in anhydrous base	1.01	100.00	99.67	Pass
		E2/E3 in aqueous base	1.01	100.00	99.28	Pass
Long term-stability	The accuracy (nominal) at each level should be ±15%	Formulation	Concentration (µg/mL)	0 day %	14 days %	Pass
		ESTROGel	1.64	100.00	99.98	Pass
		E2/E3 in anhydrous base	1.01	100.00	95.45	Pass
		E2/E3 in aqueous base	1.01	100.00	99.04	Pass

**Table 3:** Predefined acceptance criteria and results for dilution integrity of estradiol.

Acceptance Criteria	Results			
	Dilution Factor	Accuracy (%)	Precision (%)	Pass
5 replicates per dilution factor Accuracy: ±20% nominal concentrations Precision: ±20% nominal concentrations	1:5	103.50	7.24	Pass
	1:50	91.15	5.59	Pass
	1:100	87.52	4.99	Pass
	1:200	82.76	9.54	Pass
	1:300	70.52	5.43	Reject

**Table 4:** *P* values of the simple main effects of formulation and time, and their interaction effects determined by ANOVA (two-way factor with replication).

	Source of Variation	ESTROGEL Versus			
		E2/E3 Anhydrous	E2 Anhydrous	E2/E3 Aqueous	E2 Aqueous
Flux	Formulation	2.7 × 10^−9^	5.7 × 10^−10^	8.5 × 10^−8^	1.1 × 10^−8^
	Time	1.2 × 10^−6^	2.9 × 10^−7^	6 × 10^−6^	9.5 × 10^−7^
	Interaction	5.7 × 10^−9^	5.7 × 10^−9^	2.5 × 10^−7^	9 × 10^−9^
Cumulative	Formulation	6 × 10^−11^	7.5 × 10^−11^	4.4 × 10^−9^	1.1 × 10^−9^
	Time	9.6 × 10^−8^	1.8 × 10^−7^	2 × 10^−7^	5.5 × 10^−10^
	Interaction	0.079	0.088	0.12	0.0068

**Table 5:** Donor demographics.

Donor ID	Age, Years	Race	Sex	Integrity Test Results (kΩ) (Mean ± SD)
FHU-S-040822A	55	African American	Female	16.71 ± 6.56
FHU-S-042722B	59	Caucasian	Female	23.72 ± 6.41
FHU-S-011323	54	Caucasian	Female	21.86 ± 5.83

## Data Availability

The original contributions presented in this study are included in the article.
